# Polar Growth in Corynebacterium glutamicum Has a Flexible Cell Wall Synthase Requirement

**DOI:** 10.1128/mBio.00682-21

**Published:** 2021-06-08

**Authors:** Joel W. Sher, Hoong Chuin Lim, Thomas G. Bernhardt

**Affiliations:** a Department of Microbiology, Harvard Medical School, Boston, Massachusetts, USA; b Howard Hughes Medical Institute, Harvard Medical School, Boston, Massachusetts, USA; University of Massachusetts Amherst

**Keywords:** apical growth, cell wall, morphogenesis, peptidoglycan

## Abstract

Members of the *Corynebacterineae* suborder of bacteria, including major pathogens such as Mycobacterium tuberculosis, grow via the insertion of new cell wall peptidoglycan (PG) material at their poles. This mode of elongation differs from that used by Escherichia coli and other more well-studied model organisms that grow by inserting new PG at dispersed sites along their cell body. Dispersed cell elongation is known to strictly require the SEDS-type PG synthase called RodA, whereas the other major class of PG synthases called class A penicillin-binding proteins (aPBPs) are not required for this mode of growth. Instead, they are thought to be important for maintaining the integrity of the PG matrix in organisms growing by dispersed elongation. In contrast, based on prior genetic studies in M. tuberculosis and related members of the *Corynebacterineae* suborder, the aPBPs are widely believed to be essential for polar growth, with RodA being dispensable. However, polar growth has not been directly assessed in mycobacterial or corynebacterial mutants lacking aPBP-type PG synthases. We therefore investigated the relative roles of aPBPs and RodA in polar growth using Corynebacterium glutamicum as a model member of *Corynebacterineae*. Notably, we discovered that the aPBPs are dispensable for polar growth and that this growth mode can be mediated by either an aPBP-type or a SEDS-type enzyme functioning as the sole elongation PG synthase. Thus, our results reveal that the mechanism of polar elongation is fundamentally flexible and, unlike dispersed elongation, can be effectively mediated in C. glutamicum by either a SEDS-bPBP or an aPBP-type synthase.

## INTRODUCTION

Virtually all bacterial cells are surrounded by a cell wall made of peptidoglycan (PG). This heteropolymer consists of glycan chains with a disaccharide repeat and a short peptide chain attached to one of the sugars (1). The peptides are used to generate amide cross-links between glycans, which ultimately allow the formation of an interconnected meshwork that surrounds the cell and protects its membrane from osmotic rupture (1). Synthesis of the PG matrix requires two enzymatic activities: glycosyltransferases (GTases) that polymerize the glycan strands and transpeptidases (TPases) that cross-link the peptide side chains (1). The PG synthases possessing these activities come in two forms. Class A penicillin-binding proteins (aPBPs) are the most well-studied synthase type (1). They are bifunctional and possess both GTase and TPase activities in a single polypeptide (2–5). The second type of synthase was discovered more recently and is formed through an interaction between a SEDS (shape, elongation, division, and sporulation) family GTase and a monofunctional class B PBP (bPBP) with TPase activity (6–10). Despite the importance of these enzymes for cell growth and morphogenesis and their status as key antibiotic targets, we are only just beginning to understand how they work together to build and expand the cell-sized PG matrix called the sacculus that defines cell shape.

**FIG 1:**
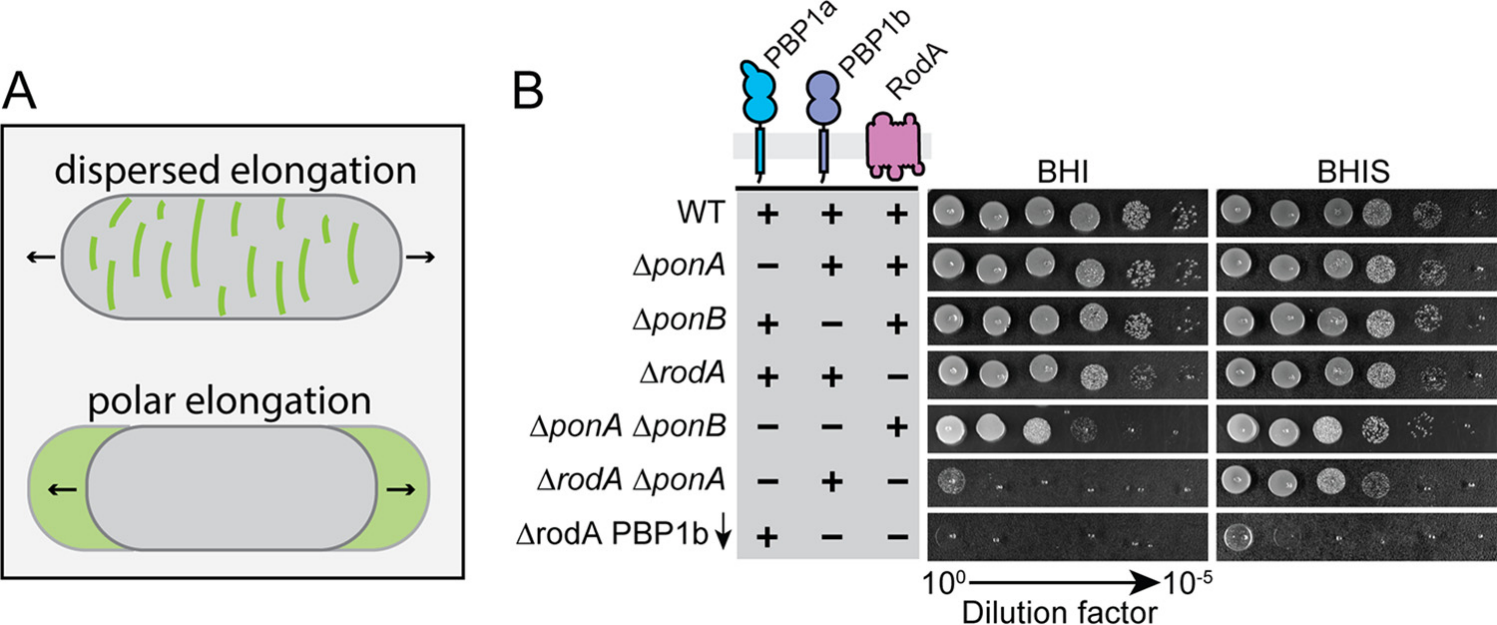
Growth phenotype of mutants inactivated for one or more elongation PG synthase. (A) Schematic highlighting the two different modes of cell elongation used by rod-shaped cells. Green color depicts areas of new cell wall insertion. (B) Overnight cultures of MB001 (WT) and its indicated derivatives HL18 (Δ*ponA*), JS20 (Δ*ponB*), HL31 (Δ*rodA*), JS36 (Δ*ponA* Δ*ponB*), JS38 (Δ*rodA* Δ*ponA*), and JS21(*attB1*::pJWS117) (Δ*rodA* Δ*ponB* [P*_sod_*::*riboE1-mScar-ponB*]) grown in BHIS medium at 30°C were normalized to an OD_600_ of 0.5 and serially diluted. An aliquot of each dilution (5 μl) was then spotted onto BHI or BHIS agar medium. Plates were incubated at 30°C for 24 h and photographed. The expression construct pJWS117 encodes an mScar-PBP1b fusion with the theophylline-inducible *riboE1* riboswitch (50) controlling its translation. Inducer was not added to the medium in order to deplete JS21(*attB1*::pJWS117) cells of PBP1b.

Bacciliform or rod shape is a common bacterial morphology. It is generated via one of two distinct modes of cell wall growth: dispersed synthesis or apical/polar elongation (11) (Fig. 1). Dispersed PG elongation has been more widely studied because it is the growth mode employed by the major Gram-negative and Gram-positive model systems, Escherichia coli and Bacillus subtilis. This growth mode is carried out by a highly conserved protein complex called the Rod system (elongasome) (1). The essential PG synthase of this system is a SEDS-bPBP complex formed between RodA and PBP2 (9). PG synthesis by this enzyme complex is spatially controlled via its connection to filaments of the actin-like MreB protein, which are thought to promote the insertion of new PG glycans at many sites around the cell cylinder in an orientation orthogonal to the long cell axis (12–14). The Rod complex also includes the membrane protein components RodZ, MreC, and MreD (15–19). Although the function of these components is still being investigated, evidence is accumulating that they are involved in modulating MreB polymerization in the case of RodZ (20) or the activation of the RodA-PBP2 synthase in the case of MreC (9). Inactivation of any of the Rod system components, including the RodA-PBP2 synthase, results in the loss of rod shape and is often lethal to organisms that grow via this dispersed mode of elongation (21).

The aPBPs are also thought to be involved in dispersed cell elongation. However, simultaneous inactivation of the two major aPBPs in E. coli and other Gram-negative bacteria results in rapid cell lysis instead of the gradual loss of shape observed when Rod system function is disrupted (22). Moreover, the aPBPs are entirely dispensable for cell growth in B. subtilis, and their activity has been associated with the production of PG material that is less oriented in structure than that produced by the Rod system (23, 24). Additionally, the localization dynamics of the aPBPs have been observed to be distinct from the proteins of the Rod system (7). Thus, the emerging picture is that the aPBPs are likely to be working outside the Rod complex and may participate in cell elongation by filling voids in the oriented PG structure produced by the MreB-directed RodA-PBP2 synthase.

Growth via the insertion of new PG material at the cell poles is an alternative mechanism by which rod shape can be generated (11). This growth mode is used by some alphaproteobacteria such as Agrobacterium tumefaciens (25). It is also the major growth mode of the *Actinobacteria* phylum, including members of the *Corynebacterineae* suborder such as Mycobacterium tuberculosis (*Mtb*), Mycobacterium smegmatis (*Msmeg*), and Corynebacterium glutamicum (*Cglu*) (26, 27). *Mtb* and its relatives lack orthologs of all Rod system components except for the RodA-PBP2 synthase. Instead of MreB, a cytoskeletal-like structure formed by the DivIVA (Wag31) protein at the cell poles is thought to organize the polar growth machinery (27–30). Although both classes of cell wall synthases localize to the cell poles (30–33), their relative roles in promoting apical growth remain unclear. In contrast to organisms that grow via dispersed elongation, RodA and PBP2 are not essential for cell elongation in the mycobacteria or *Cglu* (30, 33, 34). The essentiality of the aPBPs also varies among these organisms. In *Msmeg*, one of its two major aPBPs (PonA1/PBP1) is essential for growth, and its depletion leads to lysis and severe morphological defects (31, 35), whereas in *Mtb*, the major aPBPs are individually dispensable but form a synthetic lethal pair (36). Surprisingly, *Cglu* mutants lacking their major aPBPs were found to be viable, but the cells displayed a spherical shape (33). Based on these genetic results, polar growth in *Corynebacterineae* is generally assumed to require aPBP function. However, the process of polar growth has not been directly assessed in mutants of these organisms lacking aPBP activity. We therefore decided to reinvestigate the relative roles of the different types of PG synthases in morphogenesis and polar growth in *Corynebacterineae* using *Cglu* as a model system.

*Cglu* harbors three enzymes with PG polymerase activity that localize to the cell poles and are likely to participate in cell elongation: two aPBPs, PBP1a and PBP1b, and the SEDS protein RodA (30, 33). Surprisingly, the construction of individual and multiple deletion mutants inactivating these factors revealed that polar growth can be mediated by either type of synthase in the absence of the other. Furthermore, subcellular localization studies indicate that the aPBPs and RodA have different spatial distributions at the cell poles, suggesting that despite their individual sufficiency for polar growth, they may play distinct roles in the process. Overall, our results reveal that the mechanism of polar elongation is fundamentally flexible and, unlike dispersed cell elongation, can be effectively mediated in *Cglu* by either a SEDS-bPBP or an aPBP-type synthase.

## RESULTS

### Phenotypic analysis of *Cglu* mutants lacking a single PG synthase.

**FIG 2:**
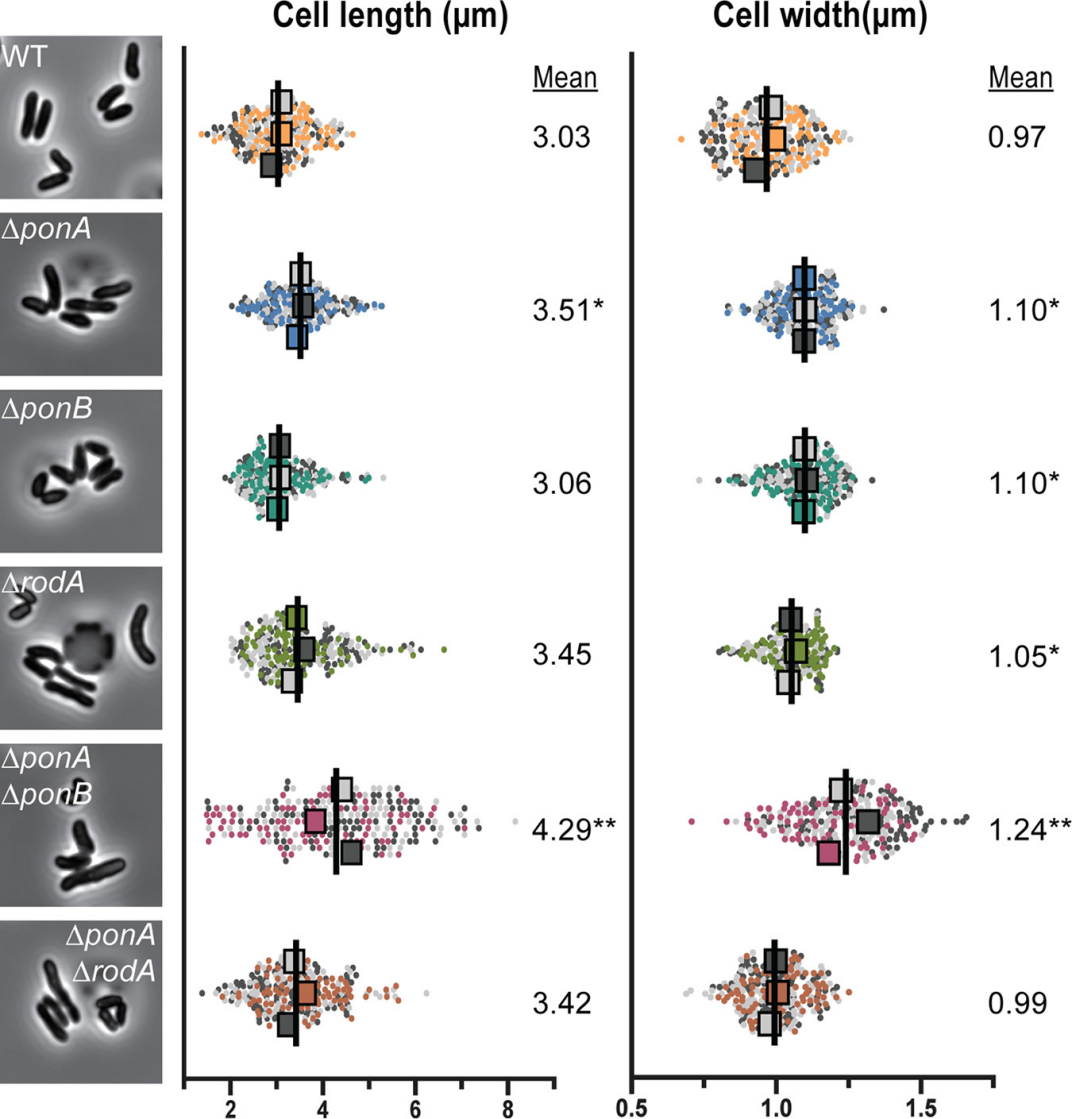
Morphological analysis of mutants lacking elongation PG synthases. Overnight cultures of the indicated strains from Fig. 1 grown in BHIS medium at 30°C were diluted 1:1,000. The cultures were then grown to an OD_600_ of 0.1 in BHIS medium at 30°C. Cells from the cultures were subsequently loaded into CellASIC devices and imaged by phase-contrast microscopy. Representative micrographs of each strain are shown adjacent to SuperPlots (51) of the corresponding morphological measurements. Cell length and width were measured for three replicate populations of 80 cells for a total of 240 cells for each strain using Oufti (48). The mean from each replicate population (represented by the large squares whose color corresponds to the replicate population from which it was calculated) was used for statistical tests. Analysis of variance (ANOVA) tests comparing replicate means to WT means were performed. *, *P* < 0.05; **, *P* < 0.0001.

To investigate the relative contributions of the different PG synthases in morphogenesis and polar growth, the corresponding genes were first deleted individually. Consistent with previous findings (30, 33), mutants lacking any single synthase were viable (Fig. 1). Deletion of either *ponA* or *ponB*, encoding the aPBP-type enzyme PBP1a or PBP1b, respectively, did not result in a substantial growth or shape defect (Fig. 1 and 2). The only observable change was that Δ*ponA* cells were slightly longer than wild-type cells (Fig. 2). A previous phenotypic profiling analysis indicated that mutants inactivated for PBP1a or PBP1b are hypersensitive to the cell wall-targeting antibiotics ampicillin or meropenem, respectively (37). These sensitivities displayed by the Δ*ponA* or Δ*ponB* mutant strains were complemented by the production of the missing synthase fused to the fluorescent protein mScarlet (mScar) (38), indicating that the phenotypes of the deletion mutants are not due to adverse effects on the expression of nearby genes (see Fig. S1A and B in the supplemental material).

10.1128/mBio.00682-21.4 FIG S1 Complementation of PG synthase mutant phenotypes. (A to C) Overnight cultures of MB001 (WT) and the indicated derivatives HL18 (Δ*ponA*), JS20 (Δ*ponB*), and HL31 (Δ*rodA*) grown in BHIS medium at 30°C were normalized to an OD_600_ of 0.5, serially diluted 10-fold, and spotted (5 μl) onto BHI or BHIS agar medium supplemented with theophylline (0.3 mM), ampicillin (0.2 μg/ml), or meropenem (0.03 μg/ml) where indicated. Plates were incubated for 24 h at 30°C and photographed. Plasmids used were pJWS19 (P*_sod_*::*riboE1-mScar-ponA Kan^r^*) and pJWS116 (P*_sod_*::*riboE1-mScar-ponA Apra^r^*] (A), pJWS94 (P*_sod_*::*riboE1-mScar-ponB Kan^r^*) and pJWS117 (P*_sod_*::*riboE1-mScar-ponB Apra^r^*) (B), and pJWS33 (P*_sod_*::*riboE1-rodA-mScar Kan^r^*) (C). Download FIG S1, TIF file, 1.8 MB. Copyright © 2021 Sher et al. 2021 Sher et al. https://creativecommons.org/licenses/by/4.0/ This content is distributed under the terms of the Creative Commons Attribution 4.0 International license.

Compared to the strains lacking one of the two major aPBPs, the *rodA* deletion mutant displayed more pronounced growth and shape defects. As observed previously, Δ*rodA* cells form smaller colonies than the wild-type control on brain heart infusion (BHI) medium at 30°C and fail to grow at 37°C (30) (Fig. 1 and S1C). However, in contrast to the prior report, which found *Cglu* cells lacking RodA to be shorter and rounder than normal (30), we observed a broad distribution of cell lengths for the Δ*rodA* mutant, with subpopulations of cells that were shorter and longer than the wild type. Overall, the average cell length of the mutant increased relative to that of the wild type (Fig. 2), and this increase in length was associated with the observation of unresolved septa in Δ*rodA* cells, indicating a defect in cell separation (see Fig. S2). A similar phenotype was previously observed for mutants lacking PBP2b (33), consistent with it being the likely bPBP partner for the RodA polymerase. Why these phenotypes are different from the previous analysis of *rodA* mutants is not clear, but it might be due to differences in the growth phase at which the cells were visualized. The shorter cells in the prior report (30) may have been imaged at a later phase of growth, potentially allowing resolution of the incomplete septa and the generation of a shorter-than-average population of cells. Nevertheless, because the phenotype of our *rodA* deletion mutant is similar to that caused by PBP2b inactivation (33) and this phenotype can be complemented by the ectopic production of a Rod-mScar fusion (Fig. S2), we conclude that cells defective for the RodA-PBP2b synthase have a cell separation defect. This finding suggests that the proper assembly of the future cell elongation machinery at the developing daughter cell pole may help complete the process of cell division (see Discussion).

10.1128/mBio.00682-21.5 FIG S2 Cell division phenotype of Δ*rodA* cells and complementation by mScar-RodA. (A) Representative phase contrast micrographs of MB001 (WT) and its HL31 (Δ*rodA*) derivative. Overnight cultures grown in BHIS medium at 30°C were diluted 1:1,000 in BHI medium and grown at 30°C. When the OD_600_ reached 0.2 to 0.3, cells were diluted 10-fold and loaded into a CellASIC microfluidic device and imaged by phase-contrast microscopy. Where indicated, RodA-mScar production was induced from integrated pJWS33 (P*_sod_*::*riboE1-rodA-mScar Kan^r^*) with theophylline (0.3 μM). The yellow arrowhead indicates longer cells within the population, while cyan arrowheads indicate unusually small cells that originated from recently v-snapped long cells. (B) Representative fluorescence micrographs of the strains from panel A grown as described above but pulsed labeled with TADA for 5 min prior to imaging. (C) Histograms showing cell length distributions of the Δ*rodA* mutant with or without RodA-mScar production. Download FIG S2, TIF file, 1.0 MB. Copyright © 2021 Sher et al. 2021 Sher et al. https://creativecommons.org/licenses/by/4.0/ This content is distributed under the terms of the Creative Commons Attribution 4.0 International license.

### Polar elongation does not require aPBP activity.

We next investigated the phenotypes of mutants lacking multiple PG synthases. Consistent with prior results (35), we were able to construct a double Δ*ponA* Δ*ponB* mutant of *Cglu* devoid of all aPBPs. Although the mutant was viable and plated with near normal efficiency on BHI medium, the resulting colonies were much smaller than those of the wild type or the mutants lacking a single aPBP (Fig. 1). Notably, the addition of sorbitol to the BHI medium (denoted as BHIS) significantly improved the growth of the double Δ*ponA* Δ*ponB* mutant, likely by osmotically protecting the cells (Fig. 1). Strikingly, when we imaged mutant cells growing under these conditions (Fig. 2) or even in plain BHI medium (see Fig. S3), their morphology was very different from that observed in the prior analysis of a double Δ*ponA* Δ*ponB* mutant, in which a single image of a small number of spherical cells was shown (35). The type of growth medium used for the prior experiment was not clearly indicated nor was a population-level analysis of cell morphology performed, but the observed spherical shape of the mutant has contributed to the commonly held belief that aPBP activity is critical for polar elongation. In contrast, our morphological analysis of Δ*ponA* Δ*ponB* mutant cells growing in BHIS medium revealed that they are quite capable of growing with a long axis, albeit with a much broader length distribution and an increased average width relative to that of wild-type cells (Fig. 2).

10.1128/mBio.00682-21.6 FIG S3 Morphology of Δ*ponA* Δ*ponB* cells grown in BHI medium without sorbitol. An overnight culture of JS36 (Δ*ponA* Δ*ponB*) grown in BHIS medium at 30°C was diluted 1:1,000 in BHI medium and grown at 30°C. When the OD_600_ reached 0.1, cells were loaded on BHI agar pads and imaged by phase-contrast microscopy. Download FIG S3, TIF file, 0.6 MB. Copyright © 2021 Sher et al. 2021 Sher et al. https://creativecommons.org/licenses/by/4.0/ This content is distributed under the terms of the Creative Commons Attribution 4.0 International license.

**FIG 3:**
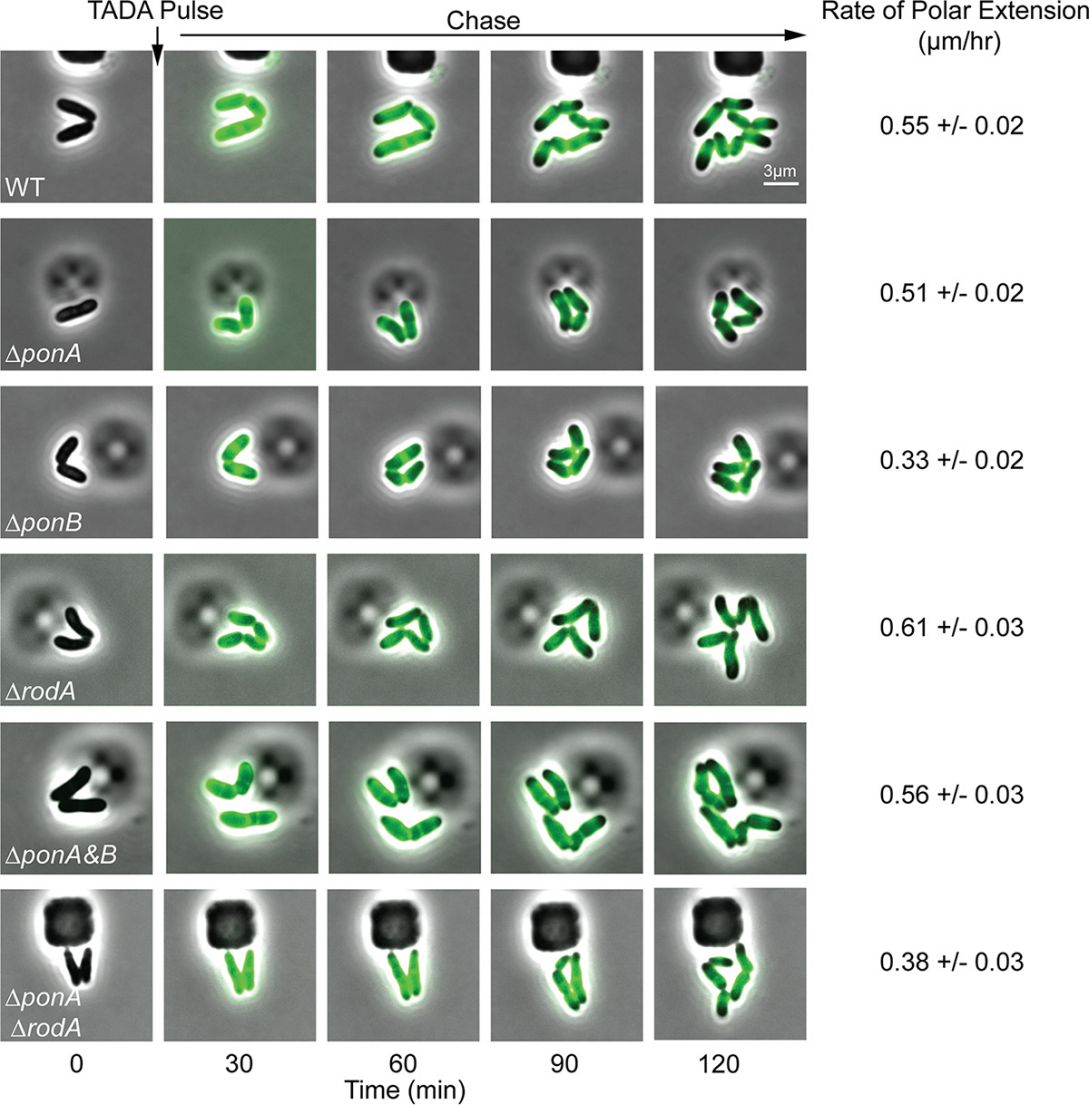
RodA and PBP1b promote cell growth by inserting nascent PG material at the poles. Overnight cultures of the indicated strains from Fig. 1 grown in BHIS medium at 30°C were diluted 1:1,000 in BHIS and grown for 3 h at 30°C. The cells were loaded into a CellASIC microfluidic device and grown for 30 min in BHIS medium at 30°C. Following this equilibration period, the cells were imaged every 5 min using phase-contrast and fluorescence optics. Cells were pulse labeled with the fluorescent d-amino acid TADA (39) for 3 min at the 6-min mark. The label was then progressively washed away by the flow of fresh medium lacking label. Every 6th frame in the time-lapse series is shown. The length of unlabeled cell wall was measured after the TADA pulse (*t* = 20 min) and at the end of the time lapse (*t* = 120 min) using Oufti (*N* > 150 cells); the unlabeled portion of each cell was defined as that with a TADA signal <20% of the maximum TADA signal for that cell. The mean and standard error of the rate of polar elongation were calculated by taking the mean difference in the length of unlabeled cell wall at the initial and final time points divided by the time elapsed.

**FIG 4:**
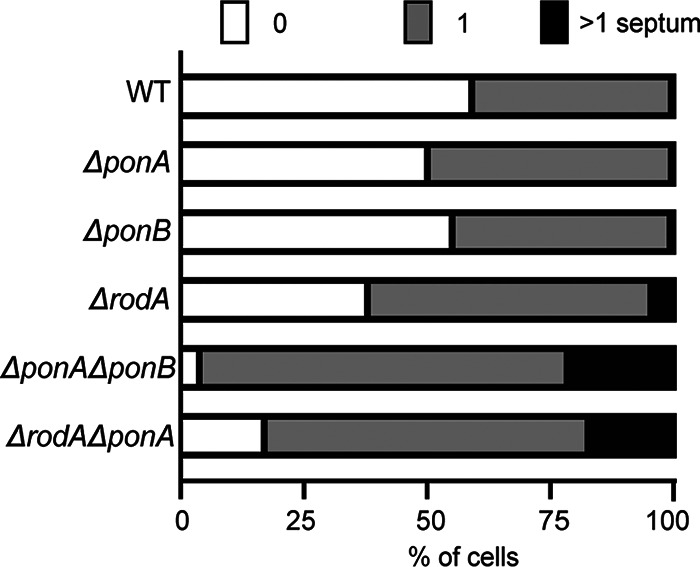
Some mutants lacking PG synthases have a cell separation defect. Using images from the 20-min time point of the time-lapse experiment in Fig. 3, the number of TADA-labeled septa in cells (*n* > 250) of each strain were quantified using the Cell Counter plugin in FIJI. The percentages of cells displaying no septa, one septum, or multiple septa are shown for each strain.

To assess the ability of the Δ*ponA* Δ*ponB* mutant to elongate from the cell pole, we performed a pulse-chase labeling experiment with the fluorescent d-amino acid (FDAA) TADA (39), which labels the PG layer (Fig. 3). Cells were first grown in the presence of TADA to label their cell walls. The label was then removed from the medium, and the cells were imaged over time by both phase-contrast and fluorescence microscopy. During this chase period, newly incorporated PG material can be monitored by the appearance of nonfluorescent areas of cell mass. As expected, for cells of the wild-type strain and mutants lacking a single PG polymerase, regions of new wall growth appeared at the cell poles (Fig. 3). The aPBP-less mutant similarly displayed the ability to grow through the addition of new PG material at the cell poles (Fig. 3). Notably, TADA labeling also revealed that this mutant had an even more severe cell separation defect than the Δ*rodA* strain, with ∼20% of cells in the Δ*ponA* Δ*ponB* population displaying multiple septa relative to ∼5% for cells lacking RodA (Fig. 4). Based on this analysis, we conclude that aPBPs are not required for polar growth in *Cglu* and that the SEDS-bPBP synthase RodA-PBP2b is likely sufficient to promote this mode of PG elongation. Additionally, the cell separation phenotype of the Δ*ponA* Δ*ponB* mutant underscores the connection between cell elongation PG synthases and the final stages of cell division in this bacterium.

### PBP1b can promote polar growth in the absence of other elongation synthases.

In addition to the Δ*ponA* Δ*ponB* mutant, we also sought to construct and characterize double mutants lacking RodA along and one of the two aPBPs. We were unable to construct either mutant on BHI medium but were successful in generating the Δ*ponA* Δ*rodA* mutant on BHIS medium. It was only possible to construct the Δ*ponB* Δ*rodA* mutant when an mScar-PBP1b fusion was ectopically expressed from a theophylline-inducible construct. Viability analysis confirmed that both the Δ*ponA* Δ*rodA* mutant and the Δ*ponB* Δ*rodA* strain depleted of PBP1b had a severe plating defect on BHI medium (Fig. 1). This plating defect was corrected on BHIS medium for the strain deleted for *ponA* and *rodA*, but even under these conditions, the mutant displayed a slow-growth phenotype relative to that of the wild type based on colony size (Fig. 1). The morphology of the Δ*ponA* Δ*rodA* mutant in BHIS medium was very similar to that of cells lacking RodA alone (Fig. 2). The cells were mildly elongated relative to wild-type cells and retained the ability to elongate from the cell poles (Fig. 3). They also displayed a cell separation defect that was increased in severity compared to that of the single *rodA* deletion mutant (Fig. 4). Because the Δ*ponB* Δ*rodA* mutant stopped growing almost immediately following PBP1b depletion, even when cultured in BHIS medium, we were unable to characterize its morphology in a manner similar to that for the other mutants in the collection.

**FIG 5:**
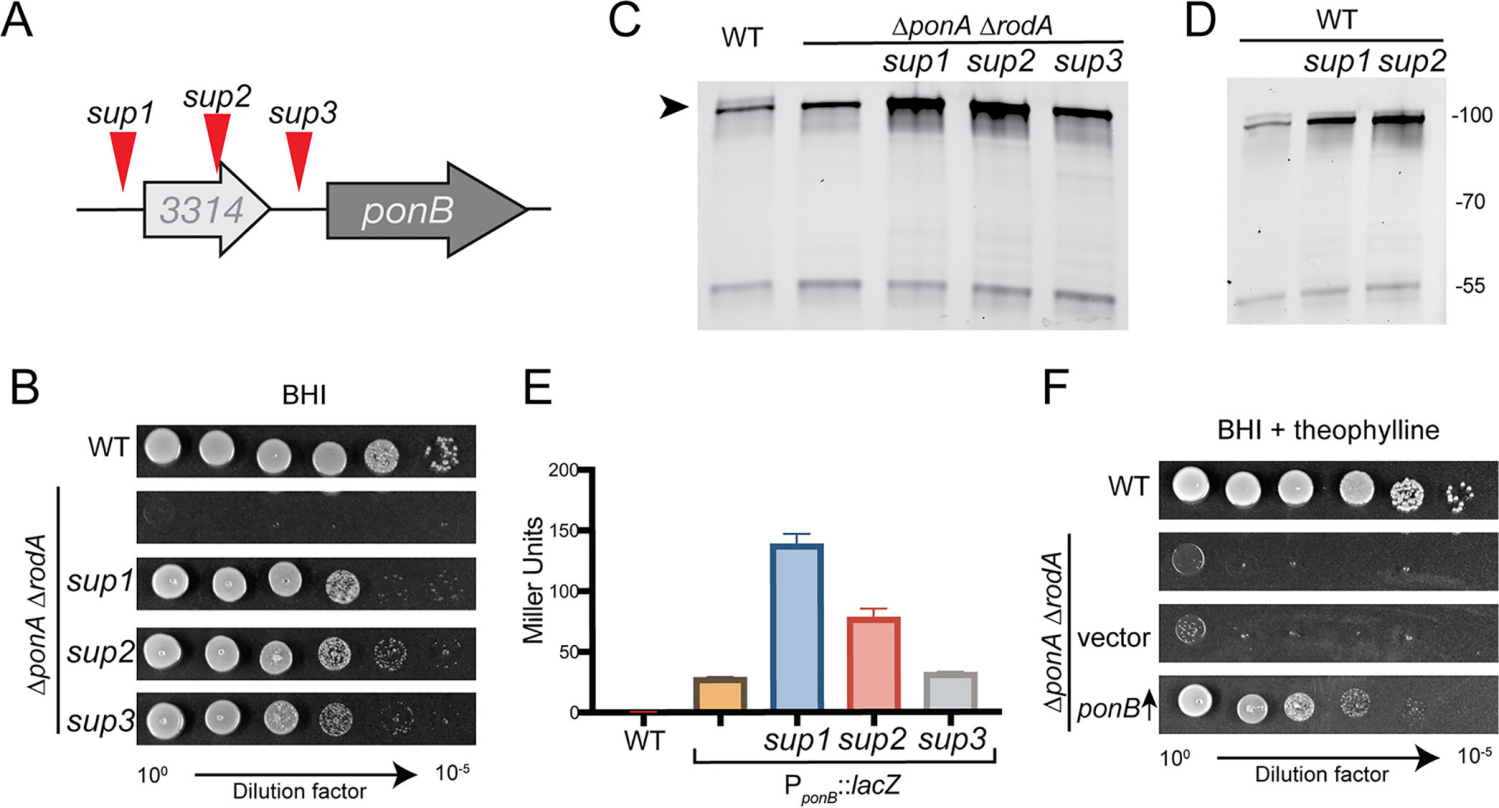
Increased PBP1b production suppresses the growth defect of Δ*rodA* Δ*ponA* cells. (A) Diagram of the *ponB* locus showing the approximate location of the suppressor mutations. The mutations mapped to nucleotide positions 2,959,545, 2,959,217, or 2,959,117 for *sup1*, *sup2*, or *sup3*, respectively, and all were G-to-A transitions. The *sup1* strain had one additional mutation in *tetA*, the *sup2* strain had no additional mutations, and the *sup3* strain had additional mutations in *fkpA*, *cgtR5*, upstream of *pitA* and *gdh*, and downstream of *phoD*. (B) Overnight cultures of the Δ*rodA* Δ*ponA* strain (JS38) and its *sup1*, *sup2*, and *sup3* derivatives were grown in BHIS medium at 30°C. Culture densities were normalized to an OD_600_ of 0.5. The normalized cultures were then serially diluted and plated as described in the legend for Fig. 1. Cells were grown on BHI medium at 30°C for 24 h before the plates were photographed. Cells of the strains from panel B (C) or MB001 (WT) and its engineered derivatives containing the *sup1* or *sup2* mutation in the *ponB* locus (D) were labeled with Bocillin-FL. Membrane extracts were then prepared from the labeled cells, and 5 μg of total protein for each was subjected to SDS-PAGE. Bocillin-FL-labeled proteins were then detected using a Typhoon 9500 imager with excitation at 488 nm and emission at 530 nm. (E) Overnight cultures of MB001 (WT) with or without a plasmid containing the indicated wild-type or mutant *ponB* locus fused to a promoterless *lacZ* reporter were grown in BHIS medium at 30°C. They were diluted 1:200 and grown to an OD_600_ of 0.3 under the same conditions. Cell pellets were then collected and frozen. Thawed cells were then resuspended in Z buffer and permeabilized with toluene and lysozyme. *o*-Nitrophenyl-β-d-galactopyranoside (ONPG) was added, CaCO_3_ was used to stop the reaction, and the absorbance of the reaction measured at 420 nm. Beta-galactosidase activity was determined using the equation activity = [OD_420_ − 1.75 (OD_550_)]/[time (min) × 1 ml vol × OD_600_ × 1,000]. (F) MB001 (WT) and its Δ*rodA* Δ*ponA* derivative with no plasmid, an empty vector, or a vector expressing mScar-PBP1b (pJWS117) were grown and plated as for panel B. The cells were grown on BHI medium supplemented with theophylline to induce PBP1b production in the pJWS117-containing strain.

To investigate why the Δ*ponA* Δ*rodA* mutant required sorbitol supplementation for growth, it was plated on plain BHI medium to isolate spontaneous suppressors that grew in the absence of sorbitol. Several survivors were purified, confirmed to have gained the ability to grow on BHI medium, and subjected to whole-genome sequencing to map the mutations causing the suppressor phenotype. Several of the isolated suppressors had one of three possible mutations near *ponB* (Fig. 5A and B). One was upstream (*sup1*) and one was within (*sup2*) the *cgp_3314* gene, which is found just upstream of *ponB*, and the third mutation (*sup3*) was located in the intergenic region between *cgp_3314* and *ponB*.

The location of the suppressor mutations upstream of *ponB* suggested that they may be affecting its expression. Accordingly, when PBP1b levels were assessed in the Δ*ponA* Δ*rodA* mutant and the suppressors using the fluorescent beta-lactam Bocillin-FL that covalently modifies the TP active site, an increase in the abundance of PBP1b was detected in the suppressor strains relative to that in the wild type and the Δ*ponA* Δ*rodA* parent strain (Fig. 5C andS4). Two of the suppressor mutations were reconstructed in an otherwise wild-type background, and both also led to an increase in PBP1b levels, indicating that the observed change does not require PBP1a or RodA to be inactivated (Fig. 5D). To determine whether the suppressor mutations were affecting *ponB* transcription, we constructed a plasmid with the region upstream of *ponB*, including *cgp_3314* and its upstream region, in front of a promoter-less *lacZ* reporter gene. Following introduction of this plasmid into the wild-type strain with or without the *sup1* or *sup2* changes at the chromosomal *ponB* locus, no change in LacZ activity was observed (not shown). However, addition of the *sup1* or *sup2* but not *sup3* changes to the plasmid resulted in an increase in LacZ activity (Fig. 5E). Thus, the suppressors only appear to be effective when they are in *cis*, with *sup1* and *sup2* causing enhanced *ponB* transcription. The remaining suppressor, *sup3*, potentially affects *ponB* translation as opposed to transcription given its limited effect on *lacZ* expression in the reporter construct.

10.1128/mBio.00682-21.7 FIG S4 Bocillin-labeling controls for determining PBP identity. Cells of MB001 (WT) and its derivatives HL18 (Δ*ponA*), JS20 (Δ*ponB*), JS12 (Δ*pbp2a*), and HL37 (Δ*pbp2b*) labeled with Bocillin-FL. Membrane extracts were then prepared from the labeled cells, and 5 μg of total protein for each was subjected to SDS-PAGE. Bocillin-FL-labeled proteins were then detected using a Typhoon 9500 imager with excitation at 488 nm and emission at 530 nm. (A) Gel was run at 110 V for 1.5 h. (B) Gel was run at 110 V for 3 h to help resolve the multiple bands at running at ∼100 kDa. Download FIG S4, TIF file, 1.6 MB. Copyright © 2021 Sher et al. 2021 Sher et al. https://creativecommons.org/licenses/by/4.0/ This content is distributed under the terms of the Creative Commons Attribution 4.0 International license.

Although the mechanism by which the suppressors are affecting *ponB* expression remains to be determined, the genetic analysis suggested that the main reason that the Δ*ponA* Δ*rodA* mutant cannot grow on BHI medium is not due to the loss of PBP1a or RodA *per se* but rather results from a general deficit in PG synthase activity. Accordingly, the overproduction of mScar-PBP1b alone was sufficient to restore growth of the Δ*ponA* Δ*rodA* mutant on BHI medium (Fig. 5F) and promote a morphology that closely matched that of the original suppressor mutants and wild-type cells (see Fig. S5). We therefore conclude that just as RodA-PBP2b appears to be sufficient to promote polar growth in the absence of the aPBPs, the aPBP-type enzyme PBP1b is also capable of promoting this mode of growth in the absence of the other elongation PG synthases. Thus, unlike dispersed cell elongation, polar elongation can be mediated by either a SEDS-bPBP synthase or an aPBP.

10.1128/mBio.00682-21.8 FIG S5 Morphology of cells with PBP1b as their only elongation PG synthase. Overnight cultures of MB001 (WT) and the indicated derivatives of JS38 (Δ*ponA* Δ*rodA*) grown in BHIS medium at 30°C were diluted 1:1,000. The cultures were then grown to an OD_600_ of ∼0.05 before being loaded into CellASIC devices for imaging by phase-contrast microscopy. Cell length and width were measured for a population of cells of each strain (*n* > 240) using Oufti. Representative micrographs of each strain are shown adjacent to plots of the corresponding morphological measurements. Each dot in the plots represents measurements from a single cell. The lines indicate the mean measurement plus or minus one standard deviation. The medium was supplemented with theophylline (0.6 mM) to induce mScar-PBP1b production in the pJWS117-containing strain. Download FIG S5, TIF file, 2.5 MB. Copyright © 2021 Sher et al. 2021 Sher et al. https://creativecommons.org/licenses/by/4.0/ This content is distributed under the terms of the Creative Commons Attribution 4.0 International license.

### PG synthase inactivation is not accompanied by increased transcription of genes encoding another synthase.

In B. subtilis, viability of the strain lacking all aPBPs depends on a stress response that increases the expression of *rodA*, presumably because excess RodA is required to compensate for the loss of the aPBP-type PG polymerases (6). This result along with our finding that suppressors of the Δ*ponA* Δ*rodA* mutant enhance *ponB* expression prompted us to investigate whether the viability of any of the deletion mutants we constructed depended on a transcriptional response that altered the expression of genes encoding the remaining PG synthases. We therefore performed transcriptome analysis (RNA-seq) on each of the deletion mutants and assessed whether the expression level for any of the genes encoding aPBP, bPBP, or SEDS proteins was altered by a deletion or pair of deletions. The only significant changes that were observed were in the expression of the deleted gene itself (Table 1), suggesting that, unlike B. subtilis, the viability of the aPBP-less strain or any other PG synthase mutant of *Cglu* does not depend on a transcriptional response that enhances the expression of other PG synthases.

**TABLE 1:** Expression of genes encoding PG synthases in mutant backgrounds

Gene	Normalized gene expression relative to WT in strains with mutation of:^*a*^				
	Δ*ponA*	Δ*ponB*	Δ*rodA*	Δ*ponA* Δ*ponB*	Δ*ponA* Δ*rodA*
*ponA*	0.001	1.010	1.096	0.001	0.001
*ponB*	1.023	0.001	1.119	0.001	1.145
*rodA*	0.993	1.022	0.001	1.135	0.002
*ftsW*	1.102	1.047	1.040	1.143	1.183
*pbpA/pbp2b*	1.042	1.028	1.367	1.145	1.575
*ftsI*	1.078	1.007	0.985	1.287	1.247
*pbp/pbp2a*	1.188	1.277	1.241	1.287	1.549

a Fold change of normalized read count for each of the listed genes in each deletion mutant relative to the read count from wild-type (MB001) cells. Raw read counts for each gene were normalized across samples to account for sample coverage. Tabulated data represent the average of duplicate RNA-seq runs for each strain.

### The different types of PG synthases have distinct polar localization patterns.

**FIG 6:**
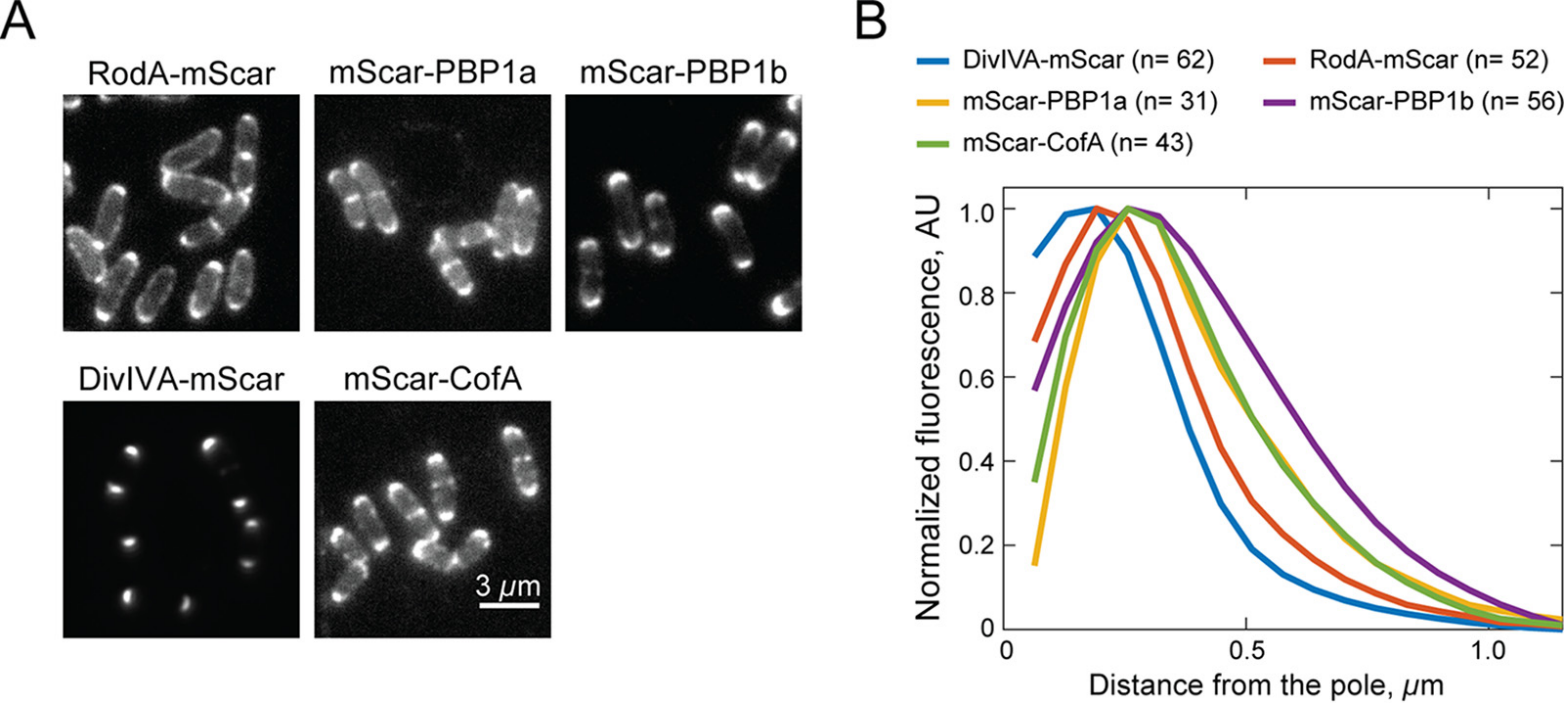
Spatial distribution of RodA and the aPBPs at the cell poles. (A) Representative fluorescence micrographs of *Cglu* cells producing the indicated mScar fusions as the sole copy of the corresponding genes either from the native locus (DivIVA-mScar, strain HL23) or integrated plasmids pJSW19 (mScar-PBP1a) in strain HL37 (Δ*ponA*), pJSW18 (mScar-CofA) in strain JS8 (Δ*cofA*), pJWS94 (mScar-PBP1b) in strain JS20 (Δ*ponB*), or pJWS33 (mScar-RodA) in strain HL31 (Δ*rodA*). Overnight cultures grown in BHI medium at 30°C were diluted 1:1,000 in BHI medium supplemented with 0.3 mM theophylline and grown at 30°C. When the OD_600_ reached 0.2 to 0.3, cells were diluted 10-fold and loaded into a CellASIC microfluidic device for imaging by fluorescence microscopy. (B) Quantification of polar fluorescence distributions of the indicated mScar fusions. Following cell segmentation by Oufti (48), a MATLAB-based script was used to identify the brightest pole of each cell, aligning the cells by setting the tip of the brightest pole to position zero. The average fluorescence intensity distribution across all cells as a function of distance from the pole was then quantified. The fluorescence profiles shown were plotted following background subtraction.

During dispersed elongation in B. subtilis and E. coli, the RodA-PBP2 and the aPBP synthases display different dynamic localizations (7), suggesting that they are performing different roles in the growth of the cylindrical cell body. We were therefore curious whether RodA and the aPBPs might have discernably different localization patterns at the cell pole of *Cglu*. Strains producing mScar fusions to RodA, PBP1a, or PBP1b as the sole copy of the corresponding synthase were constructed. All three fusion proteins were deemed functional, as they complemented the drug or heat sensitivity phenotypes of mutants deleted for the gene encoding the relevant native synthase (Fig. S1). Consistent with previous reports (30, 33), cells producing these fusion proteins all displayed prominent fluorescent signals at the cell poles and at sites of cell division (Fig. 6A). Notably, upon close examination, the patterns generated by mScar-RodA versus that of the tagged aPBPs appeared to be subtly different. The RodA-mScar signal was more concentrated at the cell tip, whereas the signals for the aPBPs appeared to encompass a greater area of the cell pole (Fig. 6A). Quantification of the fluorescence distributions along the long axis of the cell extending from the brightest pole confirmed the difference in localization patterns between RodA and the aPBPs (Fig. 6B). Notably, the distribution of RodA-mScar mirrored the tight localization of DivIVA-mScar at the polar tip, whereas the localization pattern of an mScar fusion to CofA, a PBP1a interaction partner (37), displayed a broader localization pattern similar to that of the aPBPs (Fig. 6A and B).

**FIG 7:**
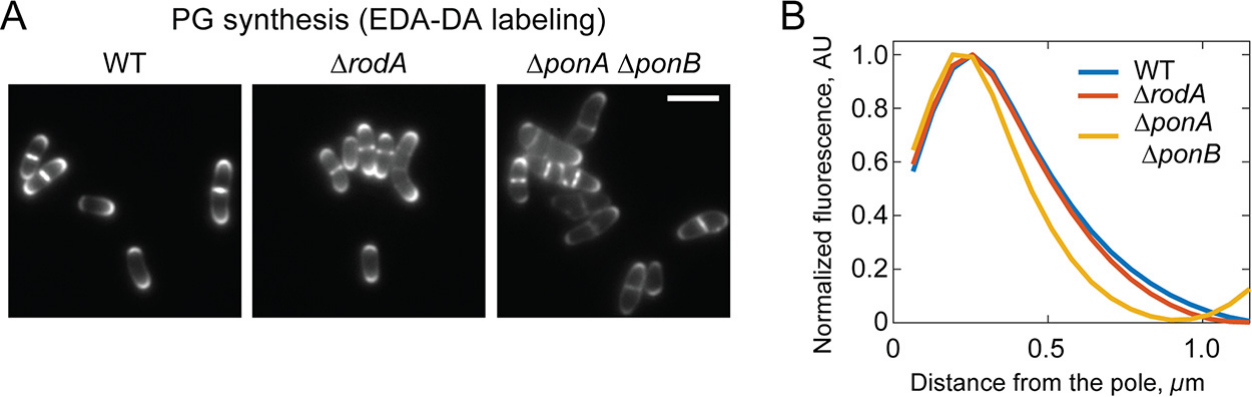
Localization of PG insertion at the poles of cells inactivated for subsets of PG synthases. (A) Representative fluorescent micrographs of MB001 (WT) and its indicated derivatives HL31 (Δ*rodA*) and JS36 (Δ*ponA* Δ*ponB*) showing incorporation of nascent PG over a period of 5 min. Overnight cultures grown in BHIS medium at 30°C were diluted 1:1,000 in BHIS medium, grown to an OD_600_ of 0.2, gently pelleted, and resuspend into BHIS medium supplemented with 0.5 mM d-Ala-d-ala dipeptide derivative EDA-DA. Cells were fixed with 70% ethanol after 5 min, followed by click chemistry labeling of the EDA moiety with Alexa 488. (B) Quantification of polar PG labeling was performed as described for Fig. 6B.

To determine if the differential spatial distribution of the PG synthases might be reflected in the pattern of PG biogenesis, we monitored the synthesis of new PG in mutants relying on either RodA or PBP1b alone for polar growth using the d-Ala-d-Ala dipeptide analog EDA-DA (40). This chemical probe is incorporated into the peptide stem of PG precursors, which are then used in the construction of the PG layer. The spatial localization of probe incorporation into the cell wall can then be assessed in fixed cells using click chemistry to couple a fluorescent dye to the EDA moiety. After a 5-min pulse with EDA-DA, Δ*rodA* cells, which rely on the aPBPs for polar growth, were found to incorporate new PG over a broader portion of the cell pole than Δ*ponA* Δ*ponB* cells, which use RodA for elongation (Fig. 7A and B). Thus, the patterns of new PG incorporation in these strains correlate well with the corresponding polar distributions observed for the type of enzyme performing the synthesis. Although more advanced microscopic methods will be required to gain a more definitive higher-resolution picture of polar PG synthesis, these results suggest that the aPBP and SEDS-bPBP synthases involved in apical elongation may have specialized roles in the process and operate from distinct polar subregions.

## DISCUSSION

In this report, we revisited the requirements of different PG synthases for polar growth in *Cglu*. Our results revealed a surprising flexibility for this mode of cell elongation and morphogenesis. Unlike the dispersed mode of PG synthesis employed by many model rod-shaped bacteria, which has a strict requirement for the SEDS-type PG polymerase RodA, polar growth and rod-shape determination in *Cglu* remains functional when either RodA or the aPBPs are inactivated. Although we cannot rule out a contribution of the SEDS-type PG polymerase FtsW to cell elongation in cells inactivated for RodA, we think this possibility is unlikely, because: (i) the overproduction of FtsW-green fluorescent protein (GFP) in *Cglu* was previously shown not to correct the growth defect of Δ*rodA* cells (30), (ii) FtsW is dispensable for cell elongation in Streptomyces coelicolor (41, 42), and (iii) FtsW was found to localize exclusively to cell division sites in *Cglu* (30). Also, in all other organisms in which it has been studied, FtsW has been shown to specifically function in cell division. Thus, our genetic and cytological analyses indicate that there is inherent plasticity in the mechanism of PG biogenesis during polar elongation that allows the process to continue in *Cglu* with either one of the two major types of PG synthase enzymes.

### Potential division of labor between RodA and the aPBPs during polar elongation.

Despite the functionality of polar elongation in *Cglu* cells lacking either RodA or the aPBPs, the mutants have a slow-growth phenotype, indicating that both types of synthases are required for optimal fitness. The different types of enzymes are therefore unlikely to serve completely redundant functions in polar PG biogenesis. Some specialization is probable. Accordingly, RodA and the aPBPs appear to have distinct localization patterns at the cell poles in *Cglu*, with RodA being more concentrated at the cell tip and the aPBPs occupying a broader distribution around the pole. Notably, PonA1 in *Msmeg*, which is the ortholog of PBP1b in *Cglu*, has also been found to have a broad distribution extending from the fast-growing old cell pole (32). Moreover, the dispensability of RodA and essentiality of PonA1 for *Msmeg* growth (31, 34, 35) provide additional support for the two types of synthases having at least some specific roles during polar PG biogenesis. What these differential roles are and whether the aPBPs function to fortify a foundational PG structure deposited by RodA-PBP2b at the pole similar to their proposed role in the dispersed mode of cell elongation will require further investigation. Nevertheless, the fact that *Cglu* can elongate without the aPBPs indicates that, in contrast to current thinking, the process of polar elongation does not fundamentally require their activity.

### Connection between cell elongation and daughter cell separation.

Cells inactivated for RodA or the aPBPs were found to be elongated and to contain multiple septa relative to wild-type cells. A similar phenotype was previously observed for mutants lacking PBP2b (33), the presumed partner of RodA in the elongation SEDS-bPBP synthase. This observation was surprising given that these cell wall synthases are thought to function predominantly in the polar elongation process. However, the cytological experiments reported here and in previous studies (30, 33) indicate that all of these factors localize at division sites as well as the cell poles. This division site recruitment of the cell elongation PG synthases coupled with the division phenotype displayed by the mutants suggests that components of the elongation machinery may actively promote the daughter cell separation process. Accordingly, PonA1 in *Mtb* has been found to interact with the PG hydrolase RipA that is involved in cell separation (31). Although it is possible that RodA or the aPBPs function as part of the divisome, another attractive possibility that warrants further investigation is that it is not just PG cleavage at the septum (43) but also the activation of PG synthesis by the elongation enzymes at division sites that provides the mechanical strain necessary to promote the extremely rapid septal cracking observed as corynebacterial and mycobacterial cells separate (44).

### Regulation of *ponB* expression.

Not much is known about the transcriptional regulation of genes encoding aPBPs in any organism. Our genetic analysis of Δ*ponA* Δ*rodA* cells identified suppressors that promote growth of the mutant by increasing *ponB* expression. These suppressors were found to contain one of three different changes in the chromosomal region just upstream of *ponB*, two of which were found to enhance its transcription. The mechanism by which these changes elicit this effect is not clear, but the observation that they only function in *cis* suggests that they may function by altering the sequence of a local promoter to strengthen it, potentially by modulating the binding of a transcription factor, or by modifying the structure and/or stability of the *ponB* mRNA. Further work will be required to differentiate between these possibilities and determine whether a stress response or a related type of regulatory system might be responsible for the underlying phenomenon.

### Conclusion.

One of the first steps toward elucidating the mechanism of a process is determining what the minimal required components are. Here, we made the surprising finding that either one of the two known types of PG synthases can effectively promote polar growth of *Cglu*. Therefore, unlike dispersed cell elongation and cell division, which have an absolute requirement for a SEDS-bPBP-type synthase, the underlying mechanism promoting apical growth in *Corynebacterineae* must be inherently adaptable such that at least in some members of this suborder either a SEDS-bPBP or an aPBP synthase can produce a form of PG capable of extending the pole and elongating the cell. A challenge for the future will be to identify the factors that contribute to the flexibility of this growth mode and whether it makes the process more resilient to stress or antibiotic challenge than other mechanisms, perhaps as a means to enhance the survival of organisms that grow apically in the competitive soil environment.

## MATERIALS AND METHODS

### Media, bacterial strains, and plasmids.

All *Cglu* strains used in the reported experiments are derivatives of MB001 (45). Strains were grown in brain heart infusion (BHI) medium (BD) that was supplemented with 91 g/liter of sorbitol (called BHIS) (Sigma) where indicated. When necessary, 15 μg/ml kanamycin (Kan) or 2.5 μg/ml apramycin (Apra) was added to the medium to select for plasmids/expression constructs. Theophylline was added where indicated at 0.3 mM unless otherwise specified. Whenever necessary, antibiotics for E. coli cultures were used at 25 μg/ml (Kan) or 50 μg/ml (Apra). Details for plasmid constructions are provided in Text S1 in the supplemental material. All strains and plasmids used are listed in Table S1 and S2, respectively.

10.1128/mBio.00682-21.1 TEXT S1 Supplemental text with plasmid construction procedures. Download Text S1, DOCX file, 0.02 MB. Copyright © 2021 Sher et al. 2021 Sher et al. https://creativecommons.org/licenses/by/4.0/ This content is distributed under the terms of the Creative Commons Attribution 4.0 International license.

10.1128/mBio.00682-21.2 TABLE S1 Strains used in this study. Download Table S1, DOCX file, 0.01 MB. Copyright © 2021 Sher et al. 2021 Sher et al. https://creativecommons.org/licenses/by/4.0/ This content is distributed under the terms of the Creative Commons Attribution 4.0 International license.

10.1128/mBio.00682-21.3 TABLE S2 Plasmids used in this study. Download Table S2, DOCX file, 0.01 MB. Copyright © 2021 Sher et al. 2021 Sher et al. https://creativecommons.org/licenses/by/4.0/ This content is distributed under the terms of the Creative Commons Attribution 4.0 International license.

### Strain construction.

For gene deletion in *Cglu*, *w*e used the pCRD206 temperature-sensitive plasmid (46). Briefly, the deletion allele with regions corresponding to approximately 750 bp upstream and downstream of the desired deletion were inserted into pCRD206. The resulting plasmid was transformed into the appropriate recipient strain. Transformants were selected and propagated on BHIS agar supplemented with Kan at 25°C. To select for clones, colonies were purified on a BHI Kan plate and incubated at 30°C for 36 h. The resulting Kan^r^ colonies were grown in BHI liquid medium at 25°C overnight. The culture was then spread on a BHIS agarose plate supplemented with 10% sucrose and grown at 30°C. The resulting colonies were replica patched on BHIS and BHIS Kan plates to identify Kan-sensitive colonies. The deletion allele was finally confirmed by diagnostic colony PCR. The above-described procedure was modified if the strain had an inducible complementation construct by adding 0.3 mM theophylline to all of the plates to induce expression of the cloned gene. Plasmid integration at the *attB1* site was performed using derivatives of the pK-PIM vector (47). Plasmids containing the desired expression construct were introduced into the recipient by direct transformation.

### Preparation of electrocompetent *Cglu*.

A stationary-phase *Cglu* culture (10 ml) was diluted into 1 liter of BHIS medium that was supplemented with 25 g glycine, 0.4 g isoniazid, and 0.1% Tween 80 and incubated with shaking at 18°C. The culture was chilled on ice for 1 h after the optical density at 600 nm (OD_600_) reached 0.5 (typically in 16 to 18 h). Cells were then collected by centrifugation at 4,000 × *g* for 20 min. The pellet was washed once with 500 ml chilled 10% glycerol and three additional times with 100 ml chilled 10% glycerol. Cell density was adjusted to an OD_600_ of 20 before use for electroporation.

### Electroporation of DNA.

Approximately 100 ng of DNA was mixed with 100 μl of electrocompetent cells in a 1-mm electroporation cuvette (Genesee Scientific). The cells were then electroporated at 1.7 kV using a MicroPulser electroporator (Bio-Rad). The cells were recovered in 1 ml BHIS medium and then immediately heat shocked for 6 min at 46°. The cells were then grown at 30° for 1 h before plating on the appropriate agar medium.

### Microscopy.

Growth conditions and staining procedures prior to microscopy are described in the figure legends. Images were cropped and adjusted using FIJI software. Measurements of cell lengths and fluorescence signals at the single-cell level were carried out using Oufti (48).

For experiments that utilized the CellASICs device, cells were loaded into the CellASIC Onix B04 microfluidic plates (Millipore Sigma) that were attached to the microscope by using a multiwell insert. Each imaging chamber was flushed with the appropriate growth medium before loading the cells. During the course of the time-lapse imaging, appropriate growth media were supplied to the cells using a constant pressure of 60 lb/in^2^. To avoid reduction of growth rate due to phototoxicity, we lowered the intensity of the excitation light using neutral density filters (at least ND8) in all experiments.

Images were obtained using a Nikon Ti inverted microscope that is fitted with a Nikon motorized stage with an OkoLab gas incubator with a slide insert attachment, an Andor Zyla 4.2 Plus scientific complementary metal-oxide semiconductor (sCMOS) camera, Lumencore SpectraX light-emitting diode (LED) illumination, Plan Apo lambda 100×/1.45 Oil Ph3 DM lens objective, and Nikon Elements 4.30 acquisition software. Images in the green and red channels were taken using Chroma 49002 and 49008 filter cubes, respectively. The microscope was maintained at 30°C using a custom-made environmental control chamber.

### RNA sequencing.

Overnight cultures of each of the strains were diluted 1:200 in BHIS medium and allowed to grow to an OD_600_ of 0.3. An aliquot of cells (1 ml) was then pelleted and frozen at −80°C. Library preparation and Illumina sequencing were performed by GENEWIZ (South Plainfield, NJ, USA). Data analysis was performed by CLC genomics workbench.

### Bocillin labeling.

Bocillin labeling was performed as described previously (37).

### Beta-galactosidase assay.

The above-indicated strains were grown to an OD_600_ of 0.3. Cells from 1 ml of culture were then pelleted and resuspended in 1 ml Z buffer (Na_2_HPO_4_-NaH_2_PO_4_ [pH 7.0], 10 mM KCl, 1 mM MgSO_4_, 50 mM beta-mercaptoethanol) with 2% toluene to permeabilize the cells. Beta-galactosidase activity was determined with permeabilized cells as described by Miller (49).

### Suppressor selection.

Eight independent colonies of the Δ*rodA* Δ*ponA* strain were grown overnight in BHIS medium. The cultures were normalized to an OD_600_ of 0.5, and 1-μl or 2-μl aliquots were plated on BHI agar, resulting in 0 to 22 suppressors on each plate. Two to three suppressors from each of the selection plates were then selected for the whole-genome sequencing. Whole-genome sequencing of the suppressors was performed as described previously (9).
