# Supplementary figures and images for: Polar Growth in Corynebacterium glutamicum Has a Flexible Cell Wall Synthase Requirement
Source: mBio. 2021 Jun 8;12(3):e00682-21. doi: 10.1128/mBio.00682-21 (PMC8262863; doi:10.1128/mBio.00682-21)

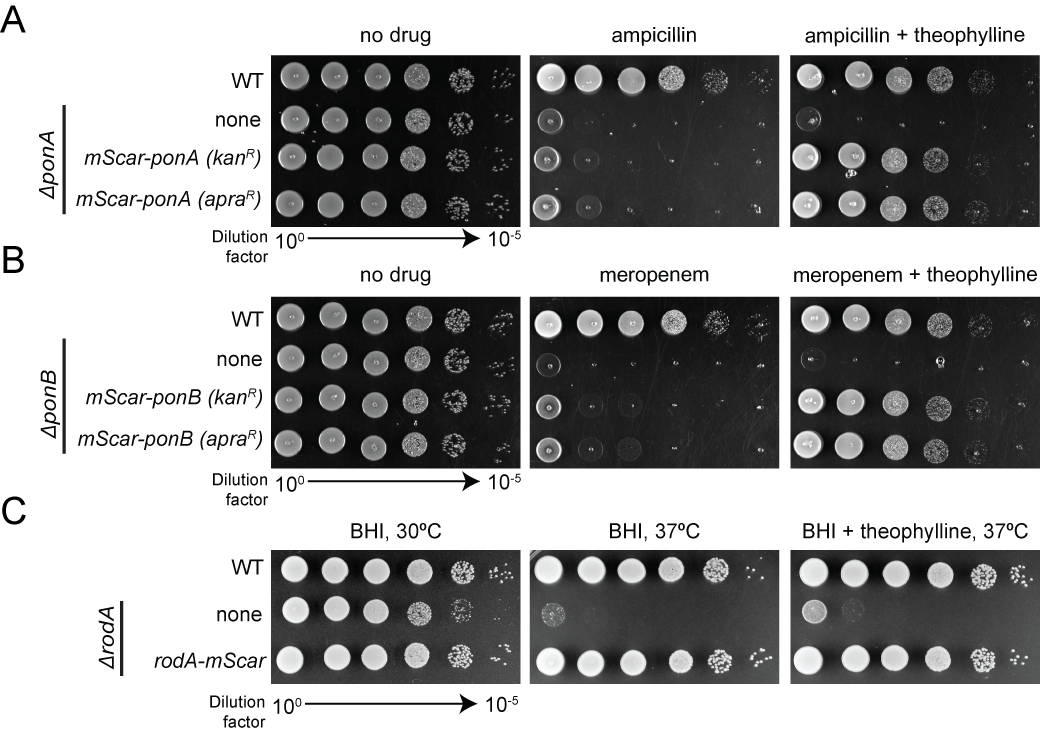

Supplement: FIG S1 [file mbio.00682-21-sf001.tif]

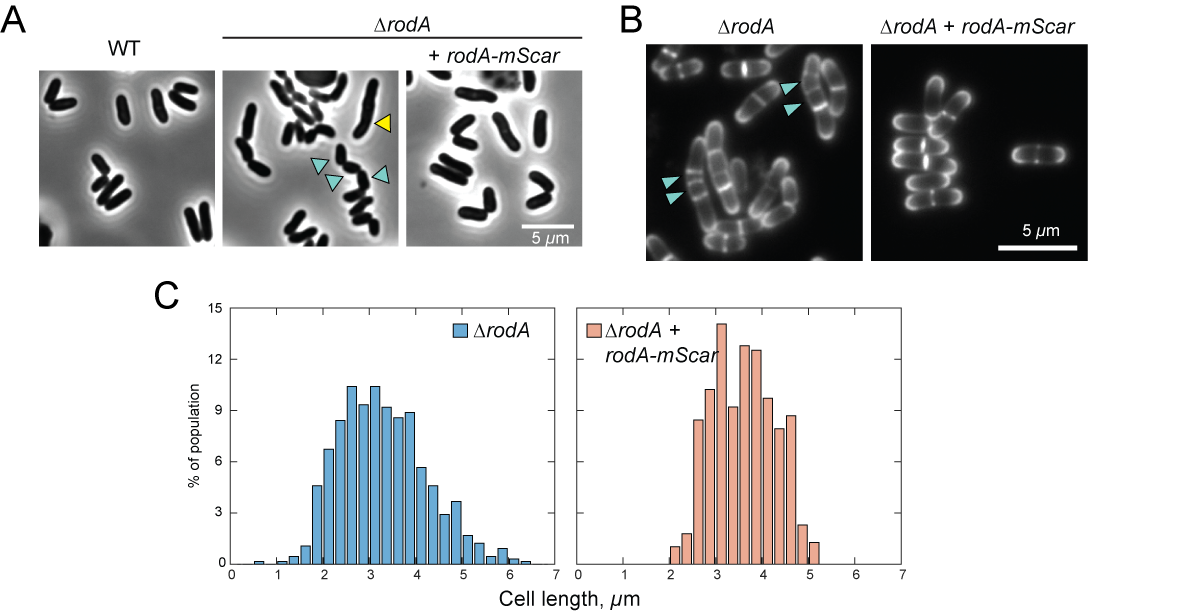

Supplement: FIG S2 [file mbio.00682-21-sf002.tif]

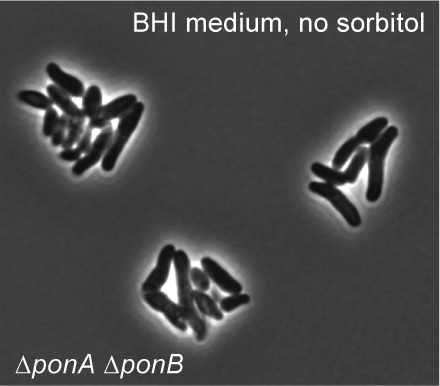

Supplement: FIG S3 [file mbio.00682-21-sf003.tif]

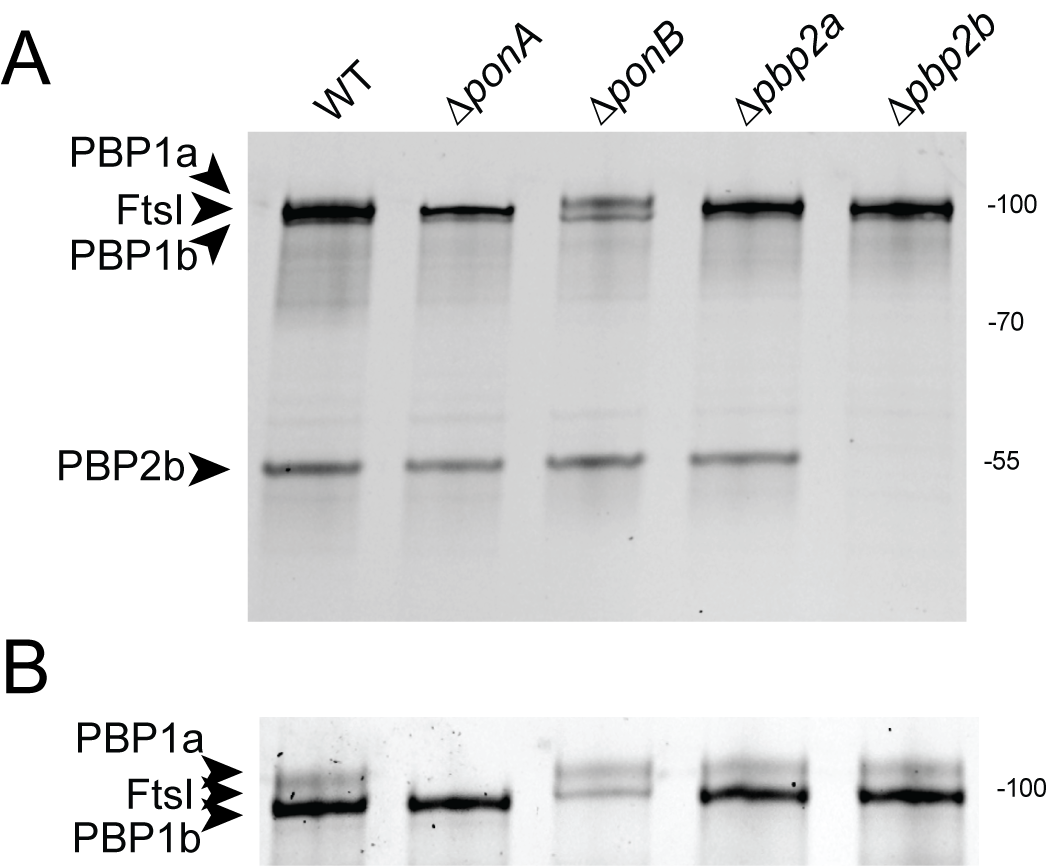

Supplement: FIG S4 [file mbio.00682-21-sf004.tif]

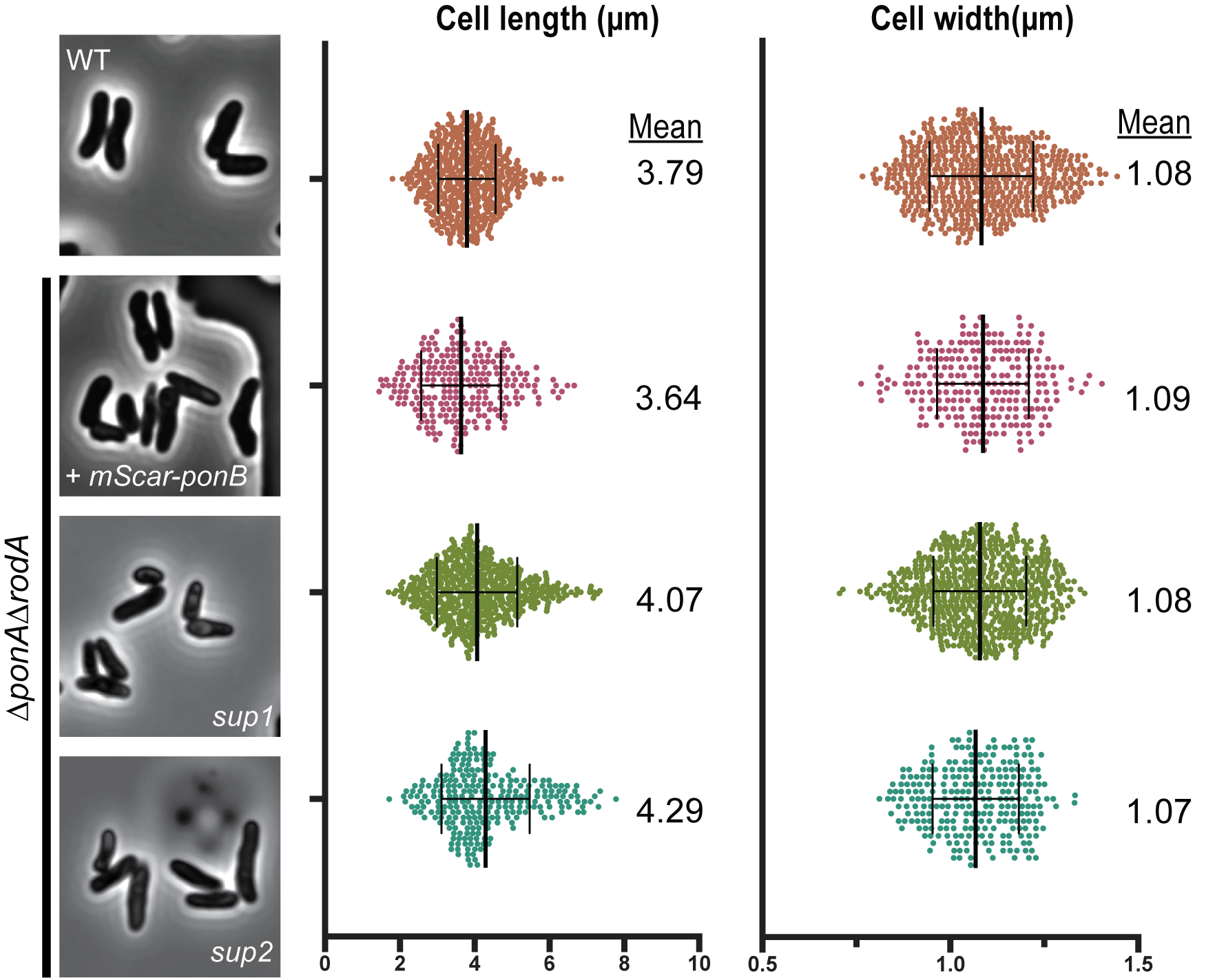

Supplement: FIG S5 [file mbio.00682-21-sf005.tif]
