# Supplementary material for: Polar Growth in Corynebacterium glutamicum Has a Flexible Cell Wall Synthase Requirement
Source: mBio. 2021 Jun 8;12(3):e00682-21. doi: 10.1128/mBio.00682-21 (PMC8262863; doi:10.1128/mBio.00682-21)
Supplement: TEXT S1 [file mbio.00682-21-s0001.docx]

**Supplemental Material For:**

**Polar growth in *Corynebacterium glutamicum* has a flexible cell wall synthase requirement**

**Authors:** Joel W. Sher^1^, Hoong Chuin Lim^1^, and Thomas G. Bernhardt^1,2^*

**Affiliations:**

^1^Department of Microbiology

Harvard Medical School

Boston, MA 02115

^2^Howard Hughes Medical Institute

*To whom correspondence should be addressed.

Thomas G. Bernhardt, Ph.D.

Department of Microbiology

Blavatnik Institute

Harvard Medical School

Boston, Massachusetts 02115

e-mail: [thomas_bernhardt@hms.harvard.edu](mailto:thomas_bernhardt@hms.harvard.edu)

**PLASMID CONSTRUCTON**

**pJWS8:** Two DNA fragments corresponding to sequences upstream and downstream of *cgp_2199 (pbp/pbp2a)* were amplified using two primer pairs: 1)
ATAAATCCTGGTGTCCCTGTTGGATCAATCGGATCGGGAACTGG & TTAGCCGACTGGCTGCTCTTCCATGGAGTGAAGATTAGCAGCG and 2)
CGCTGCTAATCTTCACTCCATGGAAGAGCAGCCAGTCGGCTAA &
CAAGCTTGCATGCCTGCAGGTCGACTGTGGAAAGTTGGGCCGATG, and MB001 gDNA as the template. The vector was amplified using AGTCGACCTGCAGGCATGCAAGCTTGGCAC &
ATCCAACAGGGACACCAGGATTTATTTATTC as the primers and pCRD206 as the template. The fragments were ligated with the vector by Gibson assembly.

**pJWS20:** Two DNA fragments corresponding to sequences upstream and downstream of *cgp_3313* (*ponB)* were amplified using two primer pairs: 1)
ATAAATCCTGGTGTCCCTGTTGGATCACAGAGCGGCGGAAATAGAG & CGTTTAGGCTACGACCGGATTTTAGCCCGTCAACTGGTGTTTCCGTTC and 2)
GATTGAACGGAAACACCAGTTGACGGGCTAAAATCCGGTCGTAGCC &
CAAGCTTGCATGCCTGCAGGTCGACTCCACAATTTGAACGCCGTAC, and MB001 gDNA as the template. The vector was amplified using AGTCGACCTGCAGGCATGCAAGCTTGGCAC &
ATCCAACAGGGACACCAGGATTTATTTATTC as the primers and pCRD206 as the template. The fragments were ligated with the vector by Gibson assembly.

**pJWS33:** NotI-digested pSEC1 was joined by Gibson assembly with the insert containing *cgp_0061* (*rodA*), which was amplified from MB001 gDNA using GAAGCTATTACCGCCGCGGCCATGAACACGCTTGAACGATTAAAGCTCC

&CGGCCAGTGAATTCACGTGCTCACGCAGCCACCTCCG as the primers.

**pJWS94:** NotI-digested pSEC1 was joined by Gibson assembly with the insert containing *cgp_3313 (ponB)*, which was amplified from MB001 gDNA using GAAGCTATTACCGCCGCGGCCTTGACGAATAGTAAAAATCCTCCTGCC

& CGGCCAGTGAATTCACGTGCTTAGCCGATCCCTAAGAGATCTCCC as the primers.

**pJWS116:** The insert encoding apramycin resistance gene was ordered as a gblock from IDT and amplified using the primers GGTTTCTTAGACGTCAGGTGGCGAAAGAAGATCCTTTGATCTTTTCTACGGGG

and CACCTAGATCCTTTTTAACCAATTCTGACCGCTCATGAGCTCAGCCA

and joined by Gibson assembly with the backbone pJWS19 amplified with TCAGAATTGGTTAAAAAGGATCTAGGTG

& GCCACCTGACGTCTAAGAAACC.

**pJWS117:** The insert encoding apramycin resistance gene was ordered as a gblock from IDT and amplified using the primers GGTTTCTTAGACGTCAGGTGGCGAAAGAAGATCCTTTGATCTTTTCTACGGGG

and CACCTAGATCCTTTTTAACCAATTCTGACCGCTCATGAGCTCAGCCA

and joined by Gibson assembly with the backbone pJWS94 amplified with TCAGAATTGGTTAAAAAGGATCTAGGTG & GCCACCTGACGTCTAAGAAACC.

**pJWS143:** Xbal and BglII-digested pSEC1 was joined by Gibson assembly with the insert containing *lacZ*, which was amplified from MG1655 gDNA using GCTAAAGGAGGTAACAACAAGATGAGATCTACCATGATTACGGATTCACTGGCC

& GACAGTTTTATTGTTCATGATGATATATTTTTATCTAGATTATTTTTGACACCAGACCAACTGGT as the primers.

**pJWS149:** SpeI and BglII-digested pJWS143 was joined by Gibson assembly with the insert containing P*_ponB_*, which was amplified from MB001 gDNA using GCTCGAGCTCTTAATTAACGTTAACTAGTAAACGATGCTTCTAGAGGGTCGT

& GGCCAGTGAATCCGTAATCATGGTAGATCTCATCTGGTGTTTCCGTTCAATCAGATCTG as the primers.

**pJWS150:** SpeI and BglII-digested pJWS143 was joined by Gibson assembly with the insert containing P*_ponB_*, which was amplified from Sup1 gDNA using GCTCGAGCTCTTAATTAACGTTAACTAGTAAACGATGCTTCTAGAGGGTCGT

& GGCCAGTGAATCCGTAATCATGGTAGATCTCATCTGGTGTTTCCGTTCAATCAGATCTG as the primers.

**pJWS151:** SpeI and BglII-digested pJWS143 was joined by Gibson assembly with the insert containing P*_ponB_*, which was amplified from Sup2 gDNA using GCTCGAGCTCTTAATTAACGTTAACTAGTAAACGATGCTTCTAGAGGGTCGT

& GGCCAGTGAATCCGTAATCATGGTAGATCTCATCTGGTGTTTCCGTTCAATCAGATCTG as the primers.

**pJWS152:** SpeI and BglII-digested pJWS143 was joined by Gibson assembly with the insert containing P*_ponB_*, which was amplified from Sup3 gDNA using GCTCGAGCTCTTAATTAACGTTAACTAGTAAACGATGCTTCTAGAGGGTCGT

& GGCCAGTGAATCCGTAATCATGGTAGATCTCATCTGGTGTTTCCGTTCAATCAGATCTG as the primers.

**pJWS159:** A DNA fragment corresponding to sequences to introduce Sup1 upstream of *cgp_3314* were amplified using the primer pairs:
ATAAATCCTGGTGTCCCTGTTGGATGTACGCGAAGGGGTTGTTTCATC & CAAGCTTGCATGCCTGCAGGTCGACTCTCGGTCTTCCGCAGCCAC and Sup1 gDNA as the template. The vector was amplified using AGTCGACCTGCAGGCATGCAAGCTTGGCAC &
ATCCAACAGGGACACCAGGATTTATTTATTC as the primers and pCRD206 as the template. The fragments were joined by Gibson assembly.

**pJWS161:** A DNA fragment corresponding to sequences to introduce Sup2 upstream of *cgp_3314* were amplified using the primer pairs: 1)
ATAAATCCTGGTGTCCCTGTTGGATGTACGCGAAGGGGTTGTTTCATC & CAAGCTTGCATGCCTGCAGGTCGACTCTCGGTCTTCCGCAGCCAC and Sup2 gDNA as the template. The vector was amplified using AGTCGACCTGCAGGCATGCAAGCTTGGCAC &
ATCCAACAGGGACACCAGGATTTATTTATTC as the primers and pCRD206 as the template. The fragments were joined by Gibson assembly.

**pHCL126:** Three DNA fragments corresponding to the 3'end of *divIVA*, the *mScarlet* gene, and the region downstream of *divIVA* were amplified using three primer pairs: 1) ATAACCAAGCTTACCTGTGGGTAAACTCCGCC & CAAGTAAAAGAAAGATTAGTTATCTTTCACCCGGCTCC, 2) GGTGCTTCGACCCAAGGACGTGGTTCGG & GAAAGATAACTAATCTTTCTTTTACTTGTACAGTTCATCCATGCCGC, and 3)

AAAATCGAATTCAGCATCCTCCAGCACCACTAAG & AACCACGTCCTTGGGTCGAAGCACCCTCACCAGATGGCTTGTTGTTG

using MB001 gDNA and gblock DNA from IDT as the template. The 3 fragments were isothermal assembled and amplified using AAAATCGAATTCAGCATCCTCCAGCACCACTAAG and ATAACCAAGCTTACCTGTGGGTAAACTCCGCC. The insert and vector, pK19mobsacB, were both digested using EcoRI and HindIII and ligated together.

**pHCL261:** Two DNA fragments corresponding to sequences upstream and downstream of *cgp_0061* (*rodA)* were amplified using two primer pairs: 1) ATAAATCCTGGTGTCCCTGTTGGATCCACGATTGAAGAAACAGTGCG & GGACATGACAGGTCGGCGCGTGCGACGAAGCTTTAATCG and 2) GATTAAAGCTTCGTCGCACGCGCCGACCTGTCATGTCCAAGC & CAAGCTTGCATGCCTGCAGGTCGACTGGTCAAGGTGGTGTTGGTGC , and MB001 gDNA as the template. The vector was amplified using AGTCGACCTGCAGGCATGCAAGCTTGGCAC & ATCCAACAGGGACACCAGGATTTATTTATTC as the primers and pCRD206 as the template. The fragments were joined by Gibson assembly.

**pHCL270:** Two DNA fragments corresponding to sequences upstream and downstream of cgp_0060 (pbpA) were amplified using two primer pairs: 1) ATAAATCCTGGTGTCCCTGTTGGATTTTGTTTACTGTTGCGGGCTAC& TGGTGCCAAATCCACCGCGGTTCACGCAGCCACCTC and 2) CAAGCTTGCATGCCTGCAGGTCGACTGATCGCCACAGACACCGAAG & GTGGCTGCGTGAACCGCGGTGGATTTGGCACCAGTGC, and MB001 gDNA as the template. The vector was amplified using AGTCGACCTGCAGGCATGCAAGCTTGGCAC & ATCCAACAGGGACACCAGGATTTATTTATTC as the primers and pCRD206 as the template. The fragments were joined by Gibson assembly.
