# Supplementary material for: Polar Growth in Corynebacterium glutamicum Has a Flexible Cell Wall Synthase Requirement
Source: mBio. 2021 Jun 8;12(3):e00682-21. doi: 10.1128/mBio.00682-21 (PMC8262863; doi:10.1128/mBio.00682-21)
Supplement: TABLE S1 [file mbio.00682-21-st001.docx]

**Table S1. Strains used in this study**

| **Strain** | **Genotype** | **Source/Reference^a^** |
| --- | --- | --- |
| *Escherichia coli* strains | | |
| DH5α(λpir) | *F– hsdR17 deoR recA1 endA1 phoA supE44 thi-1 gyrA96 relA1 Δ(lacZYA-argF)U169 ϕ80dlacZΔM15 λpir* | Gibco BRL |
| *Corynebacterium glutamicum* strains | | |
| MB001 | ATCC 13032 **Δ**CGP1 (cg1507-cg1524)  **Δ**CGP2 (cg1746-cg1752) **Δ**CGP3  (cg1890-cg2071) | Baumgart 2013 |
| HL18 | MB001 *ΔponA* | Sher 2020 |
| HL23 | MB001 *divIVA::divIVA-mScarlet* | MB001/pHCL126, 2XO, This work |
| HL31 | MB001 *ΔrodA* | MB001/pHCL261, 2XO, This work |
| HL37 | MB001 *ΔpbpA* (*pbpA* encodes PBP2b) | MB001/pHCL270, 2XO, This work |
| JS8 | MB001 *Δcgp0016* | Sher 2020 |
| JS12 | MB001 *Δpbp* (*pbp* encodes PBP2a) | MB001/pJWS8, 2XO, This work |
| JS20 | MB001 *ΔponB* | MB001/pJWS20, 2XO, This work |
| JS21(attB1:: pJWS117) | MB001 *ΔponB ΔrodA* (P*_sod_*::*riboE1-mScar-ponB*) | JS21 (attB1::pJWS117)/ pHCL261, 2XO, This work |
| JS36 | MB001 *ΔponB ΔponA* | JS20/pHCL86, 2XO, This work |
| JS38 | MB001 *ΔponA ΔrodA* | HL18/pHCL86, 2XO, This work |

^a^Marker-less in-frame deletions by double homologous cross-over (2XO) of a temperature-sensitive integrative plasmid are described using the shorthand: Parental strain/plasmid, 2XO. In all cases, plasmid loss was confirmed by selecting for Kan^S^ and sucrose^R^ clones. Deletion was confirmed by diagnostic PCR.
