# Supplementary material for: Polar Growth in Corynebacterium glutamicum Has a Flexible Cell Wall Synthase Requirement
Source: mBio. 2021 Jun 8;12(3):e00682-21. doi: 10.1128/mBio.00682-21 (PMC8262863; doi:10.1128/mBio.00682-21)
Supplement: TABLE S2 [file mbio.00682-21-st002.docx]

**Table S2. Plasmids used in this study**

| **Plasmid** | **Descriptions** | **Source/Reference** |
| --- | --- | --- |
| *Corynebacterium glutamicum (Cglu) plasmids* | |  |
| pJWS8 | Kan^R^, pCRD206 derivative containing an insert covering upstream and downstream of *cgp_2199 (pbp)* | This Study |
| pJWS18 | Kan^R^, pK-PIM derivative encoding  P*_sod_::riboE1-mscar-cgp_0016 (cofA)* | Sher 2020 |
| pJWS19 | Kan^R^, pK-PIM derivative encoding  P*_sod_::riboE1-mscar-cgp_0336 (ponA)* | Sher 2020 |
| pJWS20 | Kan^R^, pCRD206 derivative containing an insert covering upstream and downstream of *cgp_3313* (*ponB)*. | This Study |
| pJWS33 | Kan^R^, pK-PIM derivative encoding  *P_sod_::riboE1-mscar-cgp_0061 (rodA)* | This Study |
| pJWS94 | Kan^R^, pK-PIM derivative encoding  *P_sod_::riboE1-mscar-cgp_3313 (ponB)* | This Study |
| pJWS116 | Apra^R^, pK-PIM derivative encoding  *P_sod_::riboE1-mscar-cgp_0336 (ponA)* | This Study |
| pJWS117 | Apra^R^, pK-PIM derivative encoding  *P_sod_::riboE1-mscar-cgp_3313 (ponB)* | This Study |
| pJWS143 | Kan^R^, pK-PIM derivative encoding  *P_sod_::riboE1-lacZ* | This Study |
| pJWS149 | Kan^R^, pK-PIM derivative encoding  *P_ponB_-lacZ* | This Study |
| pJWS150 | Kan^R^, pK-PIM derivative encoding  *P_ponB-Sup1_::lacZ* | This Study |
| pJWS151 | Kan^R^, pK-PIM derivative encoding  *P_ponB-Sup2_::lacZ* | This Study |
| pJWS152 | Kan^R^, pK-PIM derivative encoding  *P_ponB-Sup3_::lacZ* | This Study |
| pJWS159 | Kan^R^, pCRD206 derivative containing an insert to introduce *sup1* upstream of *cgp3314* | This Study |
| pJWS161 | Kan^R^, pCRD206 derivative containing an insert to introduce *sup2* in *cgp3314* | This Study |
| pHCL86 | Kan^R^, pCRD206 derivative containing an insert covering upstream and downstream of *cgp_0336 (ponA)*. | Lim 2019 |
| pHCL126 | Kan^R^, pCRD206 derivative containing the C-terminus of DivIVA with an mScarlet fusion. | This Study |
| pHCL261 | Kan^R^, pCRD206 derivative containing an insert covering upstream and downstream of *cgp_0061* (*rodA)*. | This Study |
| pHCL270 | Kan^R^, pCRD206 derivative containing an insert covering upstream and downstream of *cgp_0060 (pbpA)* | This Study |
